# Updates on diagnostic criteria for hereditary haemorrhagic telangiectasia in the light of whole genome sequencing of ‘gene-negative’ individuals recruited to the 100 000 Genomes Project

**DOI:** 10.1136/jmg-2023-109195

**Published:** 2023-08-16

**Authors:** Claire L Shovlin, Fatma I Almaghlouth, Ali Alsafi, Nicola Coote, Catherine Rennie, Gillian MF Wallace, Fatima S Govani, Genomics England Research Consortium

**Affiliations:** 1 National Heart and Lung Institute, Imperial College London, London, UK; 2 Specialist Medicine, Imperial College Healthcare NHS Trust, London, UK; 3 Imaging, Imperial College Healthcare NHS Trust, London, UK; 4 Paediatrics, Imperial College Healthcare NHS Trust, London, UK; 5 ENT Surgery, Imperial College Healthcare NHS Trust, London, UK; 6 North Tees and Hartlepool NHS Foundation Trust, Hartlepool, UK; 7 Genomics England, London, UK

**Keywords:** Mutation, Phenotype, Genetic Linkage, Vascular Diseases

Hereditary haemorrhagic telangiectasia (HHT) is diagnosed clinically by the Curaçao Criteria of spontaneous recurrent nosebleeds, mucocutaneous telangiectasia at characteristic sites, visceral involvement (arteriovenous malformations (AVMs); gastrointestinal telangiectasia) and family history.^1^ Early diagnosis is important to enable AVM screening and preventative treatments.^2–5^ HHT is caused by loss-of-function DNA variants in *ENG*, *ACVRL1*, *SMAD4* or *GDF2*,^6–9^ though older manuscripts describing linkage to additional loci^10 11^ continue to be referenced heavily. In whole genome sequencing (WGS) performed prospectively for HHT ‘gene-negative’ patients recruited to the National Health Service (NHS) 100 000 Genomes Project,^12^ no candidate variants were identified in the *HHT3* or *HHT4* loci. ‘HHT gene-negative’ families receiving a clinical positive test result included the original HHT3 family, and a family diagnosed with a related vasculopathy (capillary malformation (CM)-AVM2^13^), due to a heterozygous variant in *EPHB4* that lies on the same chromosome as the *HHT4* locus. Clinically, we conclude that molecular testing is advisable to confirm HHT as it is possible to meet three Curaçao Criteria without having HHT. For some family members with HHT who meet three criteria ‘only’ through nosebleeds, telangiectasia and family history, a designation of ‘likely’, not ‘definite’, HHT may be preferred. Scientifically, reference to early linkage studies unsupported by confirmatory sequence identification of a causal gene is discouraged, and there is no longer evidence for an independent *HHT3* locus.

To expand, HHT is a relatively common autosomal dominant disorder where early diagnosis and intervention reduce morbidity and mortality.^2–5^ A definite clinical diagnosis is defined by three of four Curaçao Criteria, namely recurrent nosebleeds, mucocutaneous telangiectasia at characteristic sites, visceral involvement (such as gastrointestinal telangiectasia or pulmonary, hepatic, cerebral or spinal AVMs) and a positive family history (an affected first-degree relative).^1^ HHT is suspected clinically in the setting of two Curaçao Criteria,^1 5^ and recent studies indicate this can be reduced to one if the single criterion is a pulmonary AVM.^14^

The widespread introduction of clinical genetic testing for HHT and AVMs has substantially improved molecular and clinical understanding of these conditions. HHT usually results from a single, heritable loss-of-function gene variant (‘mutation’) in *ENG, ACVRL1* or *SMAD4*.^6 7^ Heterozygous loss-of-function variants in *GDF2* that encode the bone morphogenetic protein (BMP)9 ligand for the proteins encoded by *ENG* and *ACVRL1* can also cause clinically indistinguishable HHT^8^ and similar conditions.^9^ These four genes encode canonical members of BMP/transforming growth factor-β superfamily receptor serine-threonine kinase pathways. Different causal genes are now shown to distinguish individuals with HHT from ‘HHT-like’ vasculopathies that overlap phenotypically with HHT.^13–15^ The spectrum of two CM-AVM syndromes is particularly relevant since like HHT, they can cause pulmonary and cerebrospinal AVMs, while their cutaneous telangiectasia can be difficult for non-specialist clinicians to distinguish from HHT. Hence, *RASA1* (CM-AVM1^15^) and *EPHB4* (CM-AVM2^13^) are included with *ACVRL1, ENG, SMAD4* and *GDF2* on many current HHT gene panels including on the NHS National Genomic Test Directory.^14^

Restricting to the major HHT causal genes of *ENG* and *ACVRL1*, recent studies emphasise that even in adults, genetically confirmed HHT may result in few Curaçao Criteria,^14^ while HHT bleeding severity may be modified by HHT-independent DNA variants.^16^ Such nuances were not available at the time of phenotypical assessments for earlier linkage studies which mapped causal gene of families with HHT to additional loci.^10 11^ Recruitment of one of these families, and additional unsolved HHT probands/families, to the 100 000 Genomes Project,^12^ provided an opportunity to apply new insights and WGS to test evidence in continued support of HHT genes at these loci.

**Figure 1:**
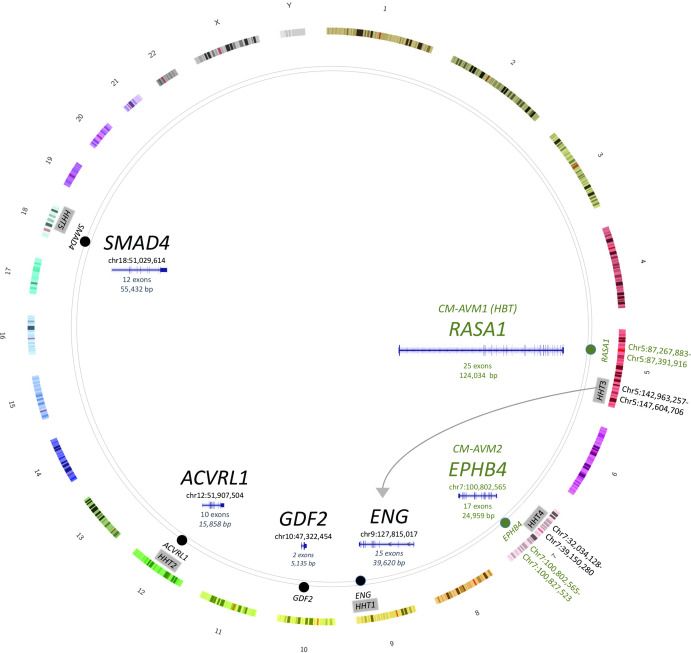
Genes and loci for HHT and CM-AVM vascular malformation syndromes. The HHT genes (*ENG, ACVRL1, SMAD4*, *GDF2*) and clinically overlapping vasculopathy genes (*RASA1, EPHB4*) plotted on a circus ideogram, with *ENG* distinguished on the reverse strand. Linkage-assigned HHT loci^10 11^ are plotted on the second ring, vasculopathy-causal genes on the third. Absolute nucleotide positions are provided for *HHT3*, *HHT4* and adjacent genes on chromosomes 5 and 7 since if drawn to scale, the very fine lines would be invisible. Inside the ideogram, for disease-causal genes, the first nucleotide is as assigned on GRCh38/hg38.^17^ The number of exons, together with the genomic structure plotted to scale, are also provided as annotated on the University of California Santa Cruz Genome Browser.^18^ CM-AVM, capillary malformation-arteriovenous malformation; HHT, hereditary haemorrhagic telangiectasia.

Briefly, *HHT3* and *HHT4* loci were assigned to human genome reference GRCh38/hg38^17 18^ using the positions of the interval-defining short tandem repeats *D5S1972-D5S490* for *HHT3* (chr5:142 963 257–147 604 706), and *D7S2252-D7S510* for *HHT4* (chr7:32 034 128–39 150 280). To visualise, HHT panel genes and the linkage intervals were plotted on an ideogram of GRCh38^17^ as described previously.^16^ Figure 1 indicates the genomic positions and exonic structures of known HHT genes, other HHT panel genes and HHT loci assigned by linkage analyses in families with HHT. Of the four linkage-assigned loci, only two have yielded HHT causal genes to date (figure 1). *ENG* was identified as the causal gene at the *HHT1* locus in 1994, and *ACVRL1* as the causal gene for *HHT2* in 1996. In contrast, casual genes for the *HHT3* 10 and *HHT4* 11 loci have not been published in the two decades since the loci were reported. No other known vascular malformation genes overlap the *HHT3* or *HHT4* loci, although *RASA1* and *EPHB4* are distantly sited on the same chromosomes as *HHT3* and *HHT4,* respectively (figure 1).

Affected members of 121 families were consented and recruited to the 100 000 Genomes Project through the West London Genomic Medicine Centre (WLGMC) if meeting the inclusion criteria for HHT (requiring three Curaçao Criteria), or pulmonary AVMs where HHT could be ‘unlikely’ (only pulmonary AVMs) or ‘suspected’ (two criteria).^14^ Curaçao Criteria were strictly applied, as originally defined: nosebleeds had to be ‘spontaneous, recurrent’^1^ and telangiectasia had to be ‘multiple, at characteristic sites (lips, oral cavity, fingers, nose)’.^1^ Visceral involvement was restricted to the specific pathologies listed in the Curacao Criteria,^1^ namely gastrointestinal telangiectasia, pulmonary AVM (the most common due to the clinical service’s referral bias^14^), hepatic, cerebral and spinal AVMs. As directed,^1^ the family history criterion was only applied if there was ‘a first-degree relative with HHT according to these criteria’. Clinically from 1999, the term ‘HHT-like’ has been used if telangiectasia appearance, sites or onset/evolution was not as expected for HHT, with such patients managed ‘as if’ HHT in terms of screening and management.

Sequencing was performed as described elsewhere^12 16 19^ and confirmed the most common molecular cause of HHT was a heterozygous loss-of-function variant in *ENG* or *ACVRL1*: Clinical Genomic Medicine Service reports indicated 65 of the recruited families had heterozygous, rare variants in either *ENG (HHT1,* N=35) or *ACVRL1 (HHT2,* N=30). Four previously ‘HHT gene-negative’ families received a positive gene test result: as reported elsewhere, a ‘first-in-family’ affected individual was found to display 15% mosaicism for an *ENG* consensus splice site variant^19^ and one family was identified as having HHT due to a *GDF2* loss-of-function missense variant.^8^ As detailed below, a pathogenic variant in *ENG* was identified in a further family with a previous negative result.

No putative candidate variants were identified in the *HHT3* or *HHT4* loci, although candidate variants were identified on chromosomes 5 and 7. A missense variant in *EPHB4* was identified in a proband who met three Curaçao Criteria, and was described as having an ‘HHT-like’ vasculopathy due to profuse, non-HHT telangiectasia present on the lips since early childhood, now recognised as typical for *EPHB4*. One of the 100 000-recruited families with HHT was found to have a missense variant of uncertain significance^20^ in *RASA1*. No other 100 000-recruited family with HHT from WLGMC was found to have a candidate variant in *EPHB4* or *RASA1*.

Importantly, a pathogenic frameshift variant was identified in *ENG* (on chromosome 9), in a member of the *HHT3* family where the HHT gene was previously reported by linkage studies to map to chromosome 5.^10^ The *ENG* variant was not present in DNA from all family members designated as affected in the earlier linkage analyses, where affected diagnoses had required three Curaçao criteria (at a minimum, recurrent nosebleeds, telangiectasia at the characteristic sites and an affected first-degree family member). No alternate candidate variants were identified in any other HHT gene. In other words, while inheritance patterns across chr5:142 963 257–147 604 706 distinguished members of this family with and without nosebleeds and telangiectasia,^10^ the region does not contain a new HHT causal gene.

Of the families who remain ‘HHT gene negative’ after recruitment through WLGMC either with HHT or with pulmonary AVMs not meeting a clinical diagnosis of HHT, eight have atypical telangiectasia (ie, not meeting typical appearances, sites or onset/evolution for HHT), three have *SMAD4*-suggestive phenotypes of aortopathies and/or gastric polyposis, while a series of additional phenotypes are evident in individual families. Wider studies are ongoing in the Respiratory GeCIP to identify causal variants.^12^

To summarise, these results indicate that phenotypical assignment of HHT can be fraught with difficulty even with pre-existing clinical expertise; that molecular diagnostics provide the most accurate route to distinguish patients and facilitate appropriate care pathways, and that there is no evidence for *HHT3*.

The important conclusion for the scientific field is that there is now no evidence for an independent *HHT3* locus.^10^ Notably, 11% of the citations to Cole *et al* 10 have been received in the past 2 years. While further detail on recent sequencing of the ‘HHT4’ family is awaited, it is suggested that reference to the old linkage papers^10 11^ should be superseded by direct sequencing data, in keeping with the progress of the field with whole exome and now WGS able to solve causality for many families where earlier, less sensitive sequencing methods did not identify a pathogenic variant.

**Figure 2:**
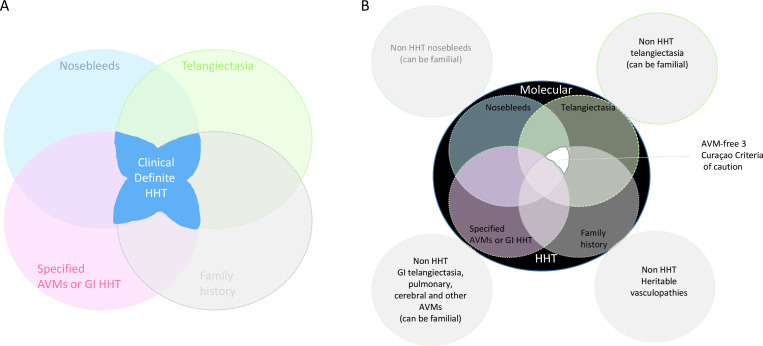
Emerging concepts in HHT clinical and molecular diagnostics. (A) The four Curaçao Criteria and combinations where three or more currently result in a definite clinical diagnosis of HHT.^1 5^ (B) Insights from new molecular data: pathogenic and likely pathogenic variants in *ACVRL1, ENG* and *SMAD4* that diagnose HHT have been identified in patients with few Curaçao Criteria (outer region of black circle). However, a diagnosis based on three Curaçao Criteria without AVMs is not specific (white region), and genetic confirmation may be encouraged. AVMs, arteriovenous malformations; GI, gastrointestinal; HHT, hereditary haemorrhagic telangiectasia.

For the clinical field, the most important finding is that the linkage to chromosome 5 reported in this journal 18 years ago following negative gene sequencing for *ENG* 10 reflected erroneous assignment of at least one positive clinical phenotype, and this has been since confirmed by further research-based sequencing. Thus, at least one of the individuals in this family clinically managed as ‘HHT affected’ meeting three Curaçao Criteria in fact is/are unaffected. The family have been informed, and further testing is being performed in the clinical sphere. We therefore suggest that alongside new awareness of the paucity of clinical signs in individuals with genetically confirmed HHT,^14^ it is also important to be cognisant of the potential lack of discrimination of recurrent nosebleeds and telangiectasia at characteristic sites in family members with HHT (figure 2). Similar comments were discussed in 1998 when the Curaçao Criteria were originally developed: HHT diagnosis at that time required only two criteria and there was concern this overdiagnosed in families with HHT, where ‘an individual may be diagnosed as affected on the basis of epistaxis alone (when epistaxis is common in the general population), or an incorrect interpretation of cutaneous vascular lesions, leading to problems in clinical management and hampering research efforts.’^1^ In 2023, the ‘HHT3’ family indicate that this potential risk of overdiagnosis can also extend to three Curaçao criteria, and that nosebleed and telangiectasia phenotypes formerly considered to represent HHT,^1 4–6^ may be present in individuals without HHT, even where one or more other family members have clear-cut, molecularly confirmed HHT.

Recognising nosebleeds are common in the general population, and with the overlapping patterns of subtle telangiectasia that may be seen in both HHT and non-HHT aetiologies, we consider an appropriate way forward would be to mirror the ‘likely pathogenic’/‘pathogenic’ distinction for variant pathogenicity.^20^ For example, where individuals in families with confirmed HHT meet the three Curaçao Criteria only through nosebleeds, telangiectasia and a first-degree affected relative, a ‘likely’ rather than ‘definite’ clinical label may be proposed. The distinction is not so important in clinical practice, because, as originally,^1^ and recently confirmed,^5^ individuals where HHT is suspected due to two criteria are also recommended for full management in order to maintain a high index of clinical suspicion and ensure appropriate screens and preventative treatments.^1 5^

Finally, the data support greater use of molecular testing within families with HHT, and may carry particular value for clinical management of a rare condition in healthcare systems where imaging and/or specialist clinicians are limiting. While frugality is not an attribute normally assigned to genetic testing, where a negative gene test removes the need for ongoing screening tests, molecular genetic testing may indeed be considered as such, enabling targeting of resources to those with the greater need. In order to maximise benefit for patients and healthcare systems:

1. For members of families where there is an existing molecular diagnosis of HHT based on a robust pathogenic or likely pathogenic variant, it seems reasonable in non-urgent settings to offer an opportunity to confirm the presence of the familial variant, before performing further AVM screening tests where an individual is asymptomatic for that type of AVM.
2. However, the converse is not appropriate. Where a molecular test for a proven HHT variant is not possible in a timely manner, is in doubt or is not patient preference, it is essential that clinical screening and investigations are not delayed, and are performed as recommended by latest consensus and local practice.

In conclusion, it was already known that HHT can be present when patients do not meet consensus clinical criteria,^14^ and this study adds that it is also possible to meet consensus clinical criteria for HHT without having HHT. The data support use of a ‘likely HHT’ designation even if three criteria are present for a relative of a patient with known HHT. Such considerations would have prevented misassignment of an *‘ENG’* HHT family’s gene location as HHT3 on chromosome 5, and we expect, will lead to wider use of HHT gene testing in clinical practice.
